# AFM study of changes in properties of horseradish peroxidase after incubation of its solution near a pyramidal structure

**DOI:** 10.1038/s41598-021-89377-z

**Published:** 2021-05-10

**Authors:** Yuri D. Ivanov, Tatyana O. Pleshakova, Ivan D. Shumov, Andrey F. Kozlov, Irina A. Ivanova, Anastasia A. Valueva, Maria O. Ershova, Vadim Yu. Tatur, Igor N. Stepanov, Victor V. Repnikov, Vadim S. Ziborov

**Affiliations:** 1 Institute of Biomedical Chemistry, Pogodinskaya str., 10, Moscow, 119121 Russia; 2 Joint Institute for High Temperatures of the Russian Academy of Sciences, Izhorskaya st. 13 Bd.2, Moscow, 125412 Russia; 3 Foundation of Perspective Technologies and Novations, Moscow, 115682 Russia; 4 Bruker Ltd., Moscow, 119017 Russia

**Keywords:** Oxidoreductases, Metalloproteins, Applications of AFM

## Abstract

In our present paper, the influence of a pyramidal structure on physicochemical properties of a protein in buffer solution has been studied. The pyramidal structure employed herein was similar to those produced industrially for anechoic chambers. Pyramidal structures are also used as elements of biosensors. Herein, horseradish peroxidase (HRP) enzyme was used as a model protein. HRP macromolecules were adsorbed from their solution onto an atomically smooth mica substrate, and then visualized by atomic force microscopy (AFM). In parallel, the enzymatic activity of HRP was estimated by conventional spectrophotometry. Additionally, attenuated total reflection Fourier-transform infrared spectroscopy (ATR-FTIR) has been employed in order to find out whether or not the protein secondary structure changes after the incubation of its solution either near the apex of a pyramid or in the center of its base. Using AFM, we have demonstrated that the incubation of the protein solution either in the vicinity of the pyramid’s apex or in the center of its base influences the physicochemical properties of the protein macromolecules. Namely, the incubation of the HRP solution in the vicinity of the top of the pyramidal structure has been shown to lead to an increase in the efficiency of the HRP adsorption onto mica. Moreover, after the incubation of the HRP solution either near the top of the pyramid or in the center of its base, the HRP macromolecules adsorb onto the mica surface predominantly in monomeric form. At that, the enzymatic activity of HRP does not change. The results of our present study are useful to be taken into account in the development of novel biosensor devices (including those for the diagnosis of cancer in humans), in which pyramidal structures are employed as sensor, noise suppression or construction elements.

## Introduction

Studying pyramidal structures attracts growing interest owing to a concentration of electromagnetic radiation near the apex and base of these structures—as was demonstrated by Balezin et al.^1^. Moreover, the use of pyramidal structures in biosensor devices was demonstrated^2^. Pyramidal structures are also employed in atomic force microscopy (AFM)-based highly sensitive sensors with probes of pyramidal shape^3^. Shielded equipment cabinets or even whole rooms, based on anechoic chambers, are widely employed in order to decrease external electromagnetic impacts on both the electronic sensors and the biological systems under study. Such chambers are fabricated on the basis of acanthoid structures, including pyramidal ones^4–6^. As a rule, these structures are organized in the form of an ordered set of pyramids, or in the form of a multilayer system of pyramids^5^. Some computational studies, considering the electric noise reduction effects of various structures, were reported^7^; therein, it was shown that a spatial change in the structure of an electric field occurs owing to both the reflection from the pyramids' surface and the damping in pyramids' material. In biological experimental studies, pyramidal elements can also be employed—for instance, as supporting or directional elements for positioning measuring cells in the sensor devices, or as sensor elements. The use of pyramidal elements can influence the spatial distribution of an electromagnetic field and, accordingly, have an effect on both the biological objects under study and the electronic measuring equipment. Thus, the influence of external electromagnetic radiation, concentrated or scattered by such pyramidal elements, on biological objects in analytical systems represents an important problem of modern biomedical research.

Herein, the influence of a single pyramidal structure on the physicochemical properties of a protein in solution has been investigated. In our experiments, a structure similar to the industrially produced pyramidal structures^8,9^, has been employed. Samples of a buffered solution of horseradish peroxidase protein were placed and incubated in various positions relative to the pyramidal structure. After the incubation, the HRP macromolecules were adsorbed from the sample solutions onto an atomically smooth surface of bare mica substrates, and visualized on this surface by AFM. The heights of the AFM images of the visualized macromolecules and their amount on the substrate surface were estimated. The use of HRP enzyme in the study was motivated by the fact that this protein is comprehensively characterized in the literature, thus being widely employed as a model in biomedical research^10^.

As was demonstrated in our previous study, atomic force microscopy (AFM) allows one to reveal the effect of electromagnetic fields on physicochemical properties of proteins—in particular, on their aggregation state and adsorbability onto solid substrate surfaces^10^. It should be emphasized that in the latter paper^10^, the electromagnetic field was generated intentionally. At the same time, electromagnetic fields of various intensities and frequencies are now extensively used in both laboratory practice and everyday life. These fields can concentrate at the expense of a resonance effect from various resonator structures, as was demonstrated by Balezin et al.^1^. These structures form specific spatial topology of electromagnetic field^1^. The effect of these external electromagnetic fields on living systems—including enzymes—can depend on the surrounding environment.

Herein, AFM has been employed to find out whether the incubation of the solution of horseradish peroxidase enzyme protein in certain locations in relation to a pyramidal structure (namely, in the vicinity of the apex of the pyramid and in the center of its base) under conventional biochemical laboratory conditions affects the protein’s properties. Alterations in the structural properties of biological macromolecules can induce changes in their functionality (one of the examples is myeloperoxidase, which is only functional in dimeric form^11^). Generally, aggregation of a protein often leads to loss in its functionality^12^. Accordingly, the results obtained are useful to be taken into account in the development of highly sensitive biosensors, in which proteins are analyzed or employed as surface-immobilized ligands. In highly sensitive biosensor systems, the measurements are performed at the level of single molecules, providing ultra-high detection sensitivity. Application of such systems will allow one to solve a number of important problems of biomedicine, including early diagnosis of diseases in humans. In this respect, however, biomedical applications of single-molecule measurements are reported in pioneer studies. Thus, all factors, including external electromagnetic fields, which potentially may influence the measurement results, must be thoroughly studied.

## Results

### Atomic force microscopy analysis

**Figure 1:**
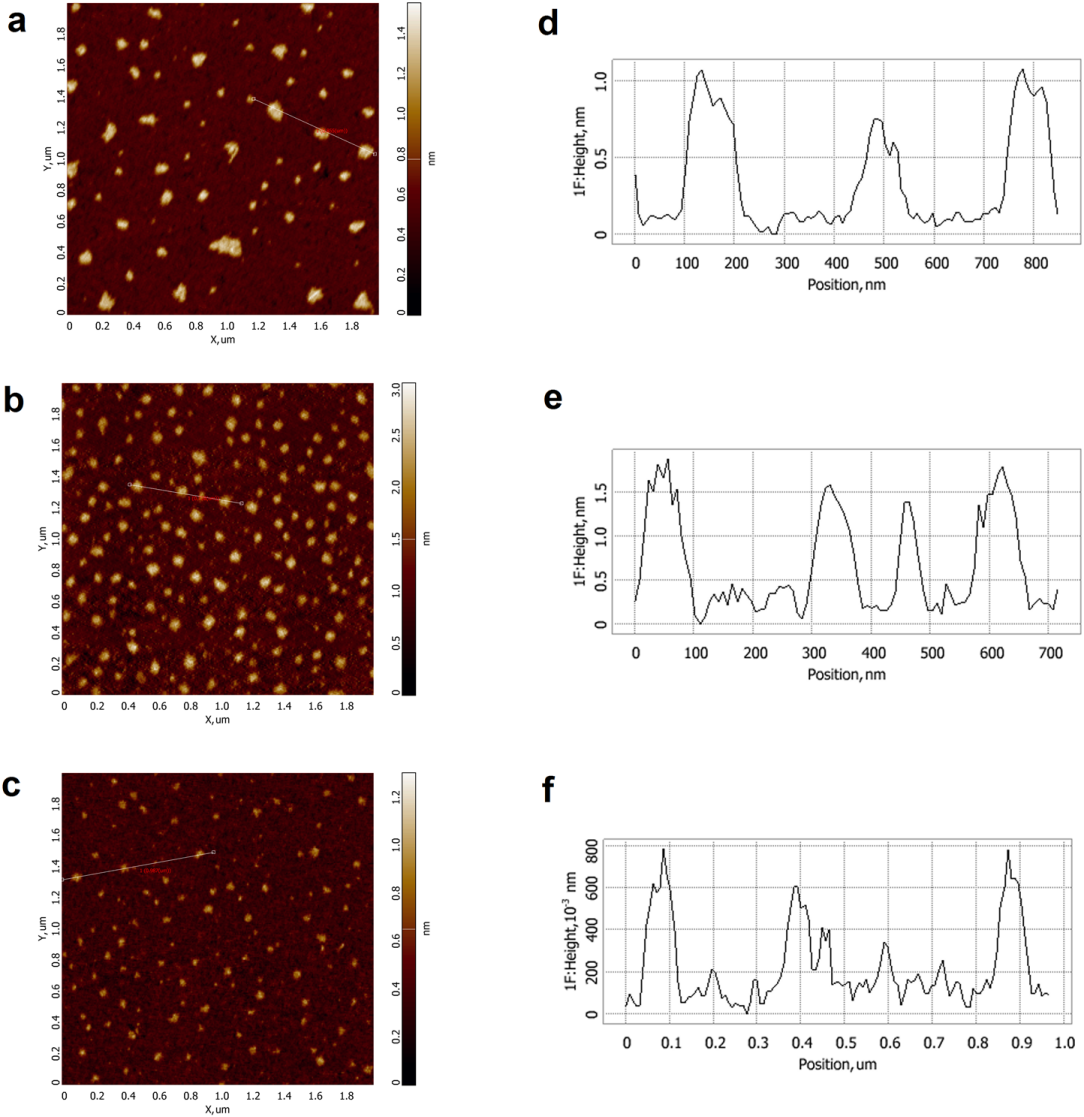
Typical AFM images (**a**–**c**) and cross-section profiles (**d**–**f**) of mica-adsorbed HRP, obtained in control experiments (**a**,**d**) and after 40-min incubation of the HRP solution in the vicinity of the apex of the pyramid (**b**,**e**) or in the center of its base (**c**,**f**). The cross-section profiles correspond to the lines shown in the respective AFM images. The images were obtained using NOVA Px software (ver. Nova_3.4_20014; NT-MDT, Moscow, Zelenograd, Russia).

Figure 1 displays typical AFM images of freshly cleaved mica surface with HRP macromolecules, adsorbed from the test solutions in control (Fig. 1a) and working (Fig. 1b,c) experiments, when the test solution was placed either in the vicinity of the pyramid's apex (Fig. 1b) or in the center of its base (Fig. 1c).

**Figure 2:**
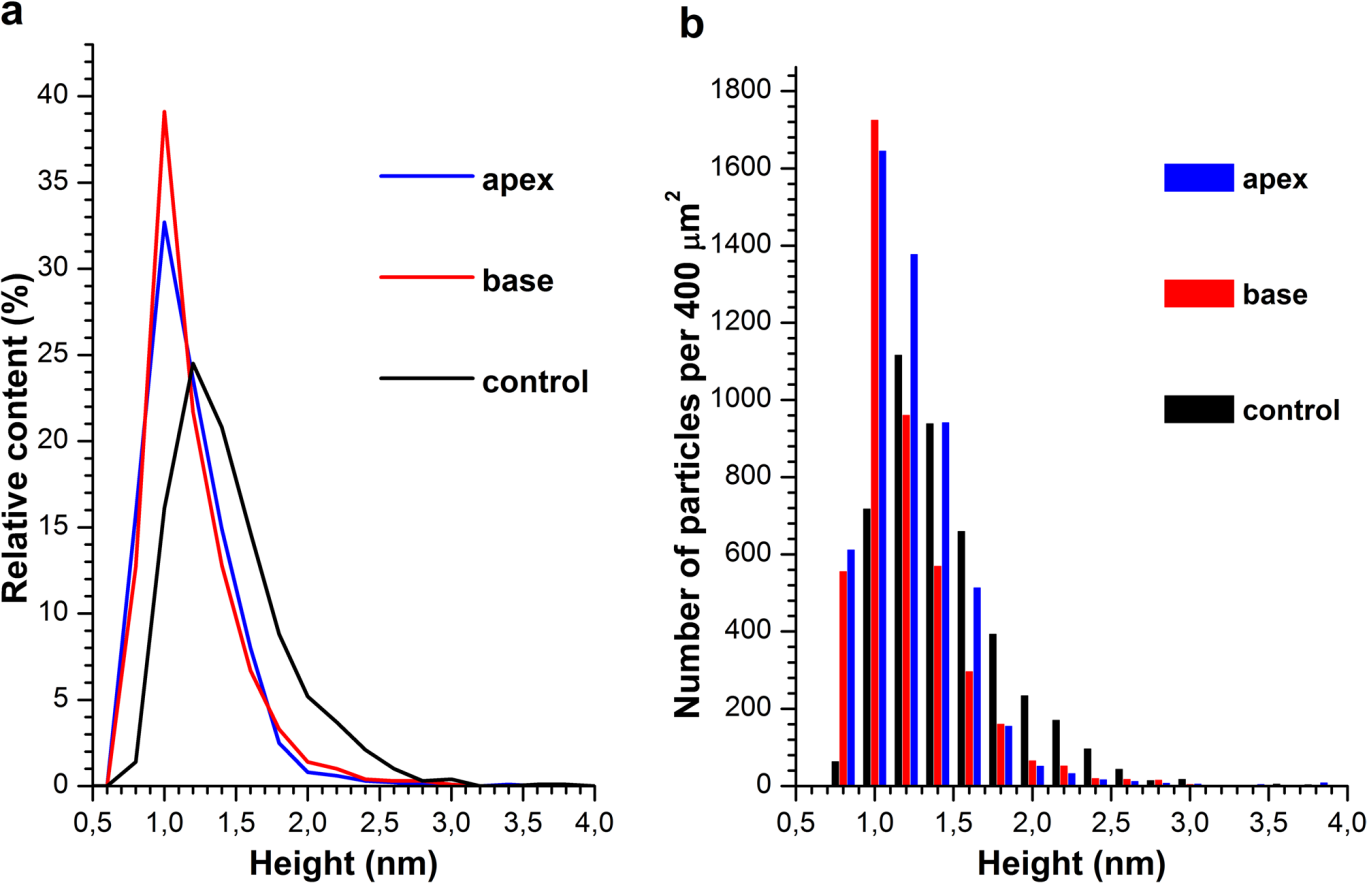
Density function *ρ*(*h*) plots (**a**) and the number of the AFM-visualized objects per 400-µm^2^ area *vs.* height (**b**) obtained in control experiments (black line and bars) and after 40-min incubation of the HRP solution in the vicinity of the apex of the pyramid (blue line and bars) or in the center of its base (red line and bars).

As seen from Fig. 1, in control and working experiments for the both cases—when the test solution was incubated either near the apex of the pyramid or in the center of its base—compact objects with heights from 1 to 2 nm were visualized on the substrate surface. Since no such objects were observed on the surface of the substrates incubated in protein-free buffer, these objects can be attributed to HRP macromolecules. After processing the AFM data obtained, the distributions of the visualized objects with height (density functions *ρ*(*h*)) were plotted. The density function plots are shown in Fig. 2a.

These *ρ*(*h*) curves indicate that in working experiments the maximum height amounts to *h*_*max*_ = (1.0 ± 0.2) nm, and in control experiments this value also makes up *h*_*max*_ = (1.2 ± 0.2) nm. That is, the density functions' maxima are equal within the measurement error. The distribution width at half-height is, however, quite different, amounting to 0.4 nm in working experiments, while in control experiments its value makes up 0.8 nm. The molecular weight of HRP is known to be 40–44 kDa^13,14^. According to the literature data, AFM images of protein molecules of similar molecular weights have heights from 1 to 1.8 nm^15–18^, depending on experimental conditions^19,20^. One can, hence, conclude that the objects of ~ 1 nm height correspond to the monomeric form of HRP, and it is the form which predominantly adsorbs onto mica in both working and control experiments. But in control experiments, broader height distribution was obtained, and the contribution of its right wing (corresponding to higher objects visualized) was more significant—in comparison with that observed in working experiments. Thus, in control samples, an increased contribution of oligomeric forms of HRP to the height distribution of the objects, visualized on mica surface, was observed.

Figure 2b displays a histogram indicating the number of objects, adsorbed onto and visualized on the mica substrate surface. One can see that the number of objects, adsorbed onto the mica surface both in control experiments and when the HRP solution was incubated in the center of the pyramid’s base, differs from that observed when the HRP solution was incubated over the pyramid’s apex. Namely, both when the test tube containing the HRP solution was placed in the center of the pyramid's base, and in control experiments, the number of HRP molecules, adsorbed onto mica in monomeric and in oligomeric forms, amounted to *N*_400_ ~ 4500 objects per 400 µm^2^ area. At the same time, in the case of placing the test tube over the pyramid's apex, this number was considerably higher, making up *N*_400_ ~ 5400 objects per 400 µm^2^.

### Results of spectrophotometric estimation of enzymatic activity of HRP

**Figure 3:**
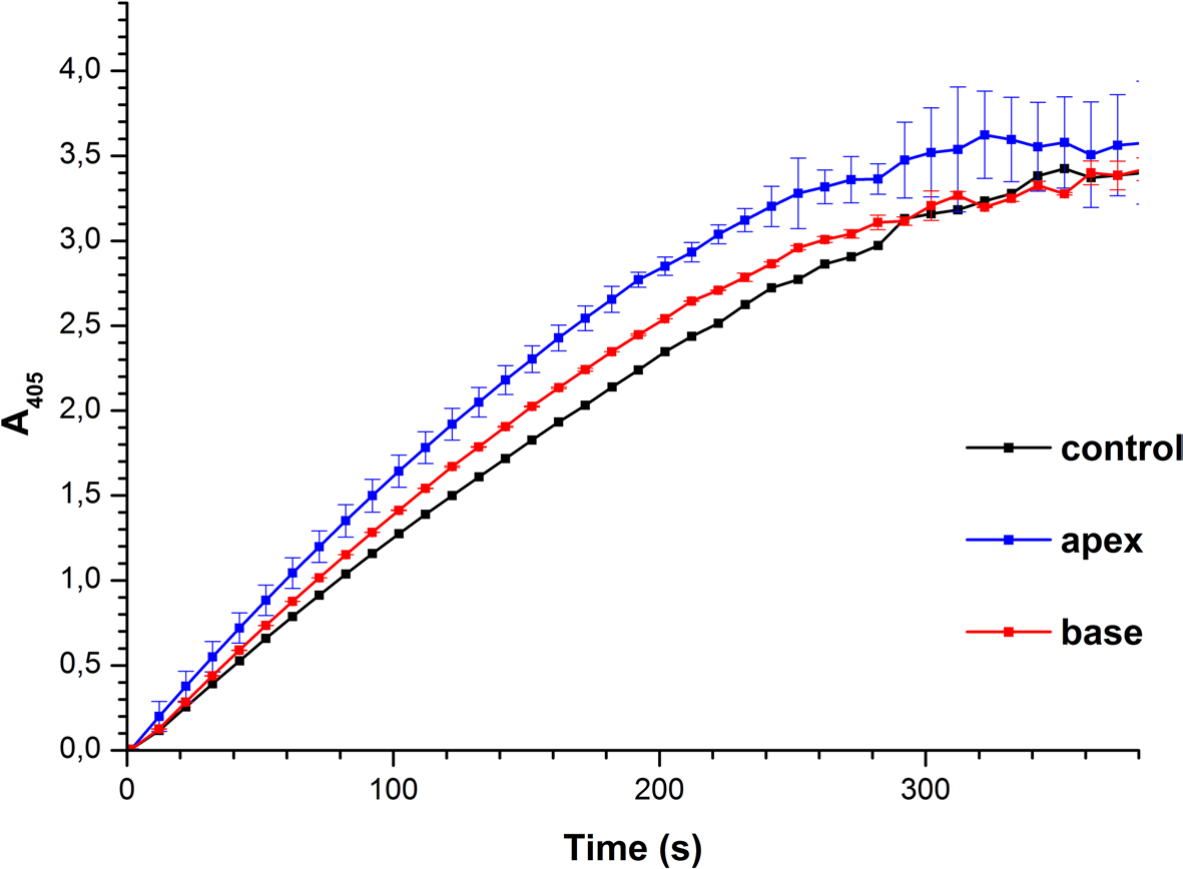
Time dependencies of the absorption at 405 nm obtained for the HRP samples in control experiments (black curve) and after the incubation of the HRP solution either near the apex of the pyramid (blue curve) or in the center of its base (red curve). Experimental conditions: HRP:ABTS:H_2_O_2_ = 10^−9^ M:0.3 mM:2.5 mM, room temperature (22 °C).

In parallel with the AFM measurements, the enzymatic activity of HRP was estimated by conventional spectrophotometry, employing the reaction with ABTS substrate in the presence of hydrogen peroxide as described in the “Methods” section. Samples treated in both working (when the test tube with the sample solution was incubated either near the apex of the pyramid or in the center of its base) and control experiments similar to those used for the AFM measurements, were studied by spectrophotometry. Figure 3 displays typical curves indicating the time dependencies of the solution absorbance at 405 nm wavelength *A*_405_(*t*), recorded for working and for control HRP solutions.

The so-obtained *A*_405_(*t*) curves shown in Fig. 3 indicate that the difference in the functional activity, observed for the HRP solutions tested, was comparable with the value of experimental error.

### Results of attenuated total reflection Fourier-transform infrared spectroscopy experiments

**Figure 4:**
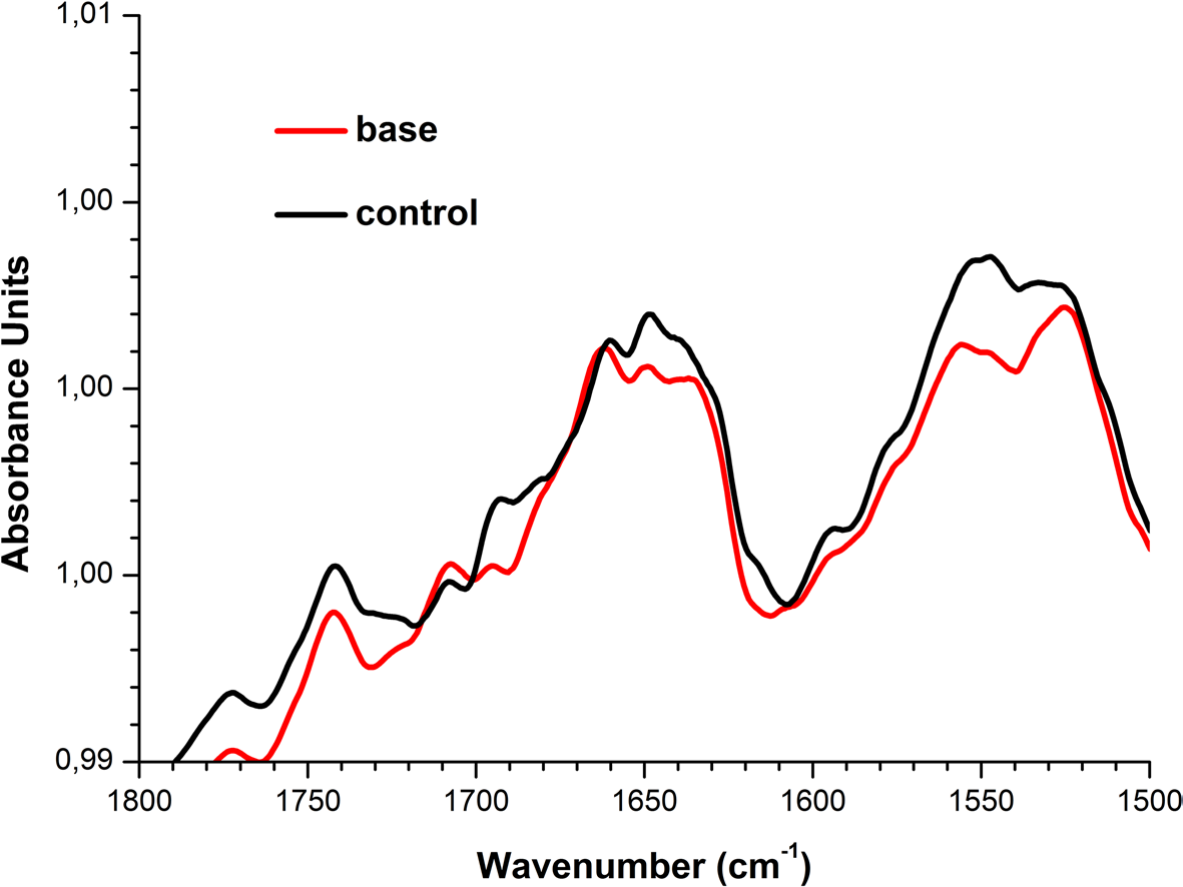
Results of attenuated total reflection Fourier-transform infrared spectroscopy (ATR-FTIR) experiments. Typical ATR-FTIR spectra obtained for 10^−4^ M HRP solutions incubated in the center of the pyramid’s base (red), or far away from the pyramid (control solution; black).

Figure 4 displays typical ATR-FTIR spectra obtained upon studying 10^−4^ M HRP solution, which was incubated in the center of the pyramid’s base, and for control HRP solution incubated at a > 10 m distance from the pyramid. In the latter case, the pyramid was suspected to have no influence on the protein solution.

Within the wavelength range from 1700 to 1500 cm^−1^, the spectrum for the control solution, shown in Fig. 4 (black curve), exhibits two characteristic peaks at 1660 cm^−1^ and 1550 cm^−1^, corresponding to Amide I and Amide II, respectively^21^. Within the wavelength range studied, no change in the ATR-FTIR spectrum of the solution incubated near the pyramid's apex was observed (data not shown)—in contrast to the case with the solution incubated in the center of the pyramid's base. Namely, for the solution incubated in the center of the base, the intensity of the 1550 cm^−1^ peak (Amide II band) is considerably lower in comparison with that obtained for the control solution. That is, the ratio between the intensities of Amide I and Amide II peaks has changed. This indicates that incubation of HRP solution in the center of the pyramid’s base leads to an alteration in the protein secondary structure.

## Discussion

As was reported by Ignatenko et al., in aqueous solutions, HRP is present in the form of a mixture of monomers and aggregates^22^. Our results obtained herein indicate that the incubation of HRP solution in the vicinity of the pyramid's apex leads to an increase in the efficiency of adsorption of the protein molecules onto the mica surface. An increased contribution of objects, whose heights are characteristic for monomeric HRP, to the height distribution of AFM-visualized objects shown in Fig. 2a, also indicates an increase in the content of the HRP macromolecules, adsorbed onto mica in monomeric form after the incubation of the HRP solution both in the vicinity of the pyramid's apex and in the center of its base. At that, the enzymatic activity of HRP does not change.

Thus, in the case of the HRP solution incubated over the pyramid's apex, changes in both the number of the adsorbed objects and the degree of the protein oligomerization were observed, while only the oligomerization degree changed in the case of the HRP solution incubated in the center of the pyramid's base —in comparison with the parameters obtained for the control samples incubated far away from the experimental setup.

The changes in adsorption properties of the biological macromolecules indicate alterations in their structure. The adsorption of proteins onto solid surfaces is known to be primarily driven by electrostatic interactions between the charged protein macromolecules and the charged surface^23,24^, though other types of interactions (such as hydrophobic ones) can also have a certain (sometimes governing) effect on protein adsorbability^25^. In our case, the adsorption of HRP onto mica represents a consequence of electrostatic interaction between the negatively charged groups of the mica surface with the surface groups of the protein globules. The change in the spatial structure of the HRP protein apparently leads to changes in the distribution of charge over the surface of the protein globule. At that, these changes do not affect the active site of HRP, as otherwise a change in its enzymatic activity would be observed. Accordingly, the strength of the interactions of the HRP molecules both between each other and with the substrate surface has changed. This change in the protein structure is expected to be connected with alterations in the intensity of external electromagnetic field near the pyramid in standard laboratory conditions.

According to ATR-FTIR data, a slight change in the secondary structure of HRP has been observed after the incubation of its solution in the center of the pyramid's base. These results correlate with significant changes in adsorption properties of the protein in this sample. According to AFM data obtained for the sample incubated in the center of the pyramid's base, the number of mica-adsorbed particles with *h* ≥ 1.4 nm decreased—in comparison with the control sample (Fig. 2b). No change in the ATR-FTIR spectrum of the solution incubated near the apex has been observed (data not shown). According to the AFM data, less significant change in the height distribution of the mica-adsorbed objects has been observed for the solution incubated near the apex of a pyramid—in comparison with that obtained for the control solution. That is, alterations in HRP secondary structure, revealed by ATR-FTIR, are in agreement with changes in the adsorption properties of HRP particles with *h* ≥ 1.4 nm (which are attributed to aggregates) in comparison with the control protein solution (Fig. 2b).

The change in the protein structure is presumably caused by an increased concentration of electromagnetic fields in the vicinity of the pyramid's apex. Recent theoretical studies^7^ revealed that this effect is more likely to occur near the pyramid's base at wavelengths, comparable to the length of the pyramid's edges. Moreover, the use of pyramidal structures, fabricated from gold, in experimental studies for concentrating the electromagnetic waves in the vicinity of the apex of near-field probes was reported^3^. The concentration of external electromagnetic fields can occur owing to a resonance effect from resonator structures, which form specific spatial topology of electromagnetic field^1^.

It should be noted that the technique involving AFM measurements in air, indeed, is not free from limitations, and the changes in protein properties measured in liquid can be somewhat different from those measured in air^15,26^; this should be studied in the future and presented in the form of a separate research. In our present manuscript, we just have tried to reveal the fact of the influence of a pyramidal structure on the properties of a protein dissolved in water—in order to draw attention of scientists working with equipment, which contains pyramidal structures (such as biosensors), to this effect. The results of our present study can be of use in the development of highly sensitive biosensors, since changes in the structural properties of biological macromolecules can also lead to a change in their functionality. The results obtained herein should also be taken into account when conducting direct measurements using biosensors equipped with pyramidal construction elements, located in the vicinity of measuring cells.

## Conclusions

The effect of pyramidal structures on the properties of a protein in buffered aqueous solution has been revealed by atomic force microscopy using horseradish peroxidase (HRP) heme-containing enzyme as a model. An increase in adsorbability of HRP macromolecules from its buffered aqueous solution onto bare mica after the incubation of this solution in the vicinity of the pyramid's apex has been demonstrated. Moreover, an increased contribution of the monomeric form of HRP to the height distribution of the mica-adsorbed objects has been observed after the incubation of the HRP solution either over the apex of the pyramid or in the center of its base—in contrast to the control HRP solutions incubated far away from the pyramid. The results obtained herein can be of use in the development of biosensor devices, and should be taken into account upon performing direct measurements with biosensors containing pyramidal elements, as well as upon studying the influence of electromagnetic radiation on biological systems.

## Methods

### Chemicals and protein

HRP protein was purchased from Sigma (Cat. #6782). 2,2′-azino-bis(3-ethylbenzothiazoline-6-sulfonate) (ABTS) was purchased from Sigma. Disodium hydrogen orthophosphate (Na_2_HPO_4_), citric acid and hydrogen peroxide (H_2_O_2_) were purchased from Reakhim (Moscow, Russia). 2 mM Dulbecco's modified phosphate buffered saline (PBSD buffer) was prepared by dissolving a certain amount of salt mixture (Pierce, USA) in water. All solutions were prepared using deionized ultrapure water (with 18.2 MΩ × cm resistivity) obtained with a Simplicity UV system (Millipore, Molsheim, France).

### Experimental setup

**Figure 5:**
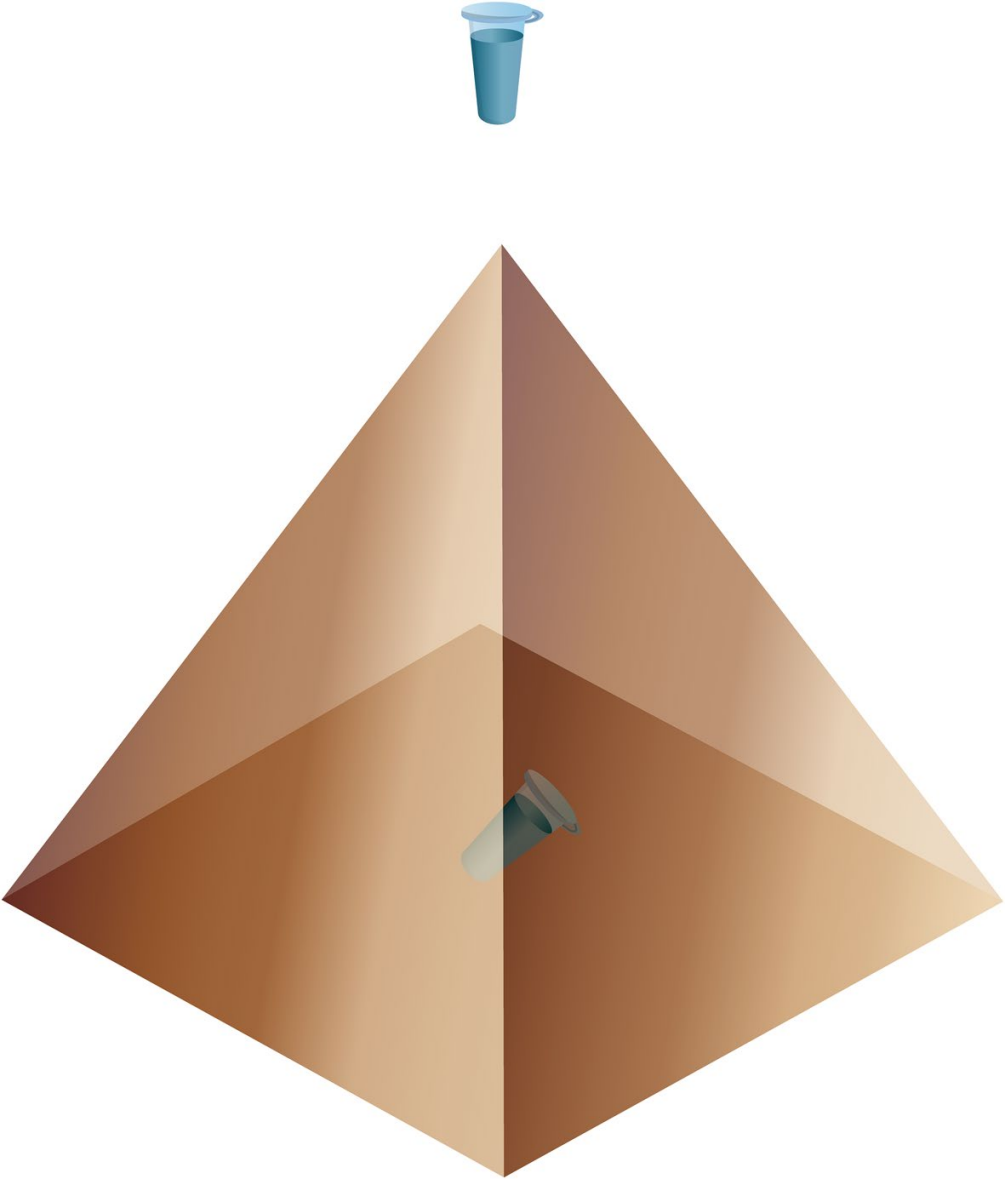
Experimental setup. The test tube with the HRP solution was placed either at 1 cm distance from the pyramid's apex, or in the center of its base.

Figure 5 displays the experimental setup employed in our study.

The 130-mm-high pyramid had the side apex angle of 52 degrees and was fabricated from 2-mm-thick copper-coated textolite sheets. An 1.7-mL Eppendorf-type polypropylene test tube (SSBiological, USA) containing 1 mL of the test HRP solution was placed in locations shown in Fig. 5—namely, either at a 1 cm distance from the pyramid's apex or in the center of its base. In control experiments, the test tube with the solution was placed far away (at a > 10 m distance, but in the same room) from the experimental setup. The test solutions were incubated within the experimental setup for 40 min. After the incubation, the test solutions were analyzed by both AFM and spectrophotometry as described below.

### Sample preparation for AFM experiments

The experiments were carried out using the direct surface adsorption method^27^. Mica sheets (SPI, USA) were used as substrates with hydrophilic surface. Upon the preparation of the AFM samples, a 7.5 × 15 mm piece of the freshly cleaved mica was immersed into 800 µL of 0.1 μM test solution of HRP in 2 mM PBSD buffer, and incubated therein for 10 min in a Thermomixer Comfort shaker (Eppendorf, Germany) at 600 rpm and room temperature. In the course of the incubation, HRP macromolecules adsorbed onto the mica surface. After the incubation, each mica substrate was rinsed with ultrapure water and then dried in air.

### Atomic force microscopy measurements

The AFM experiments were carried out as described in our recent papers^10,19,20,28^. The mica surface with adsorbed HRP molecules was visualized by AFM-based in tapping mode in air at 25 °C and 60% relative air humidity employing a Titanium multimode atomic force microscope (which pertains to “Avogadro” large-scale research facilities; NT-MDT, Zelenograd, Russia) equipped with NSG10 cantilevers (“TipsNano”, Zelenograd, Russia; 47–150 kHz resonant frequency, 0.35 to 6.1 N/m force constant). The calibration of the microscope by height was carried out on aTGZ1 calibration grating (NT-MDT, Russia; step height 21.4 ± 1.5 nm).

The main advantages of imaging in air are its simplicity and high throughput^26^, which allow us to perform more measurements in one working day without loss of the imaging quality. For these considerations, imaging in air has been employed. It is to be emphasized that the AFM measurements were performed at ~ 60% air humidity, when both the mica substrate and the studied protein are covered by a hydration layer^27,29^. That is, the conditions of our AFM experiments can be considered as near-native ones.

The total number of imaged objects in each sample was no less than 200, and the number of frames for each sample was no less than 10. Analogously to Ref.^30^, the relative density of distribution of the imaged objects with height *ρ*(*h*) was calculated as

1 $$\rho \left( h \right) = \left( {N_{h} /N} \right) \times 100\% ,$$

where *N*_*h*_ is the number of imaged proteins with height *h*, and *N* is the total number of imaged proteins^30^.

To compare the data obtained from different experiments, the number of registered objects was normalized per 400 μm^2^ area. Accordingly, the number of visualized objects was calculated using the equation

2 $$N_{{{\text{NORM}}}} = N \times {4}00/\left( {n \times {4}} \right),$$

where *N*_*NORM*_ is the number of objects per 400 μm^2^, and *n* is the number of the obtained scans (the area of each scan is 2 × 2 = 4 μm^2^).

Preliminary experiments were performed with the use of protein-free buffer solution instead of protein solution; in these experiments, no objects of > 0.5 nm height was registered. AFM operation, obtaining AFM images, their treatment (flattening correction etc.) and exporting the obtained data in ASCII format were performed using the standard NOVA Px software (ver. 3.4_20014; NT-MDT, Moscow, Zelenograd, Russia) supplied with the atomic force microscope. The number of the visualized particles in the obtained AFM images was calculated automatically using specialized AFM data processing software developed in Institute of Biomedical Chemistry.

### Spectrophotometric estimation of enzymatic activity of HRP

HRP activity was estimated using ABTS as reducing substrate, as described in our previous papers^10,19,28^, employing the technique reported by Sanders et al.^31^. ABTS is commonly employed for the determination of HRP enzymatic activity, and the enzymatic assay reaction should be performed at pH 5.0^32^. Briefly, the rate of change in solution absorbance at 405 nm was measured employing an Agilent 8453 UV–visible spectrophotometer (Agilent Technologies Deutschland GmbH, Waldbronn, Germany). Then, 30 μL of 10^−7^ M HRP solution was added into a 3-mL quartz cuvette (pathlength 1 cm, Agilent, USA) containing 2.96 mL of 0.3 mM ABTS solution in phosphate-citrate buffer (51 mM Na_2_HPO_4_, 24 mM citric acid, pH 5.0) and stirred. In this way, the final HRP concentration in the cuvette was 10^−9^ M. Finally, 8.5 μL of 3% (w/w) H_2_O_2_ was added into the cuvette. Spectrum acquisition was started immediately upon the addition of H_2_O_2_.

### Attenuated total reflection Fourier-transform infrared spectroscopy

In order to find out whether or not the HRP secondary structure changes after the incubation of its solution either near the apex of a pyramid or in the center of its base, ATR-FTIR method was employed. The ATR-FTIR measurements were carried out in the following way. 12 µL of 10^−4^ M solution of HRP in PBSD was placed in the measuring cell of an INVENIO spectrometer (Bruker Scientific LLC, Billerica, MA, USA). Such a high concentration of the protein solution was used due to the sensitivity of the spectrometer. The data were presented in a standard form provided by the spectrometer operation software. In order to account for the contribution from the PBSD buffer to the resulting spectra, a blank measurement with pure protein-free buffer was performed before the experiments with HRP solutions within the same spectral range. The spectrum obtained in the blank measurement was subtracted from those obtained in experiments with HRP solutions.
